# Effects of using different levels of flaxseed in dairy cattle diet on production performance and milk fatty acid profile

**DOI:** 10.1007/s11250-025-04446-z

**Published:** 2025-04-25

**Authors:** Yusuf Koçoğlu, Hayati Köknaroğlu, Sakine Yalçın

**Affiliations:** 1 Department of Animal Science, Isparta University of Applied Sciences, Isparta, Türkiye; 2 Faculty of Veterinary Medicine, Department of Animal Nutrition and Nutritional Diseases, Ankara University, Ankara, Türkiye

**Keywords:** Dairy cattle, Flaxseed, Milk fatty acid profile

## Abstract

The purpose of this study was to determine the effects of using different levels of flaxseed in dairy cattle diet on performance and milk fatty acid profile. For this purpose, 9 lactating Holstein cows of the same parity and lactation stage were used. The study followed a crossover design with three periods, three treatment groups, and three cows per group. Each period lasted 21 days, of which 14 days was the adaptation period and 7 days was the data collection period. During the experiment, 2 different concentrate feeds were used, along with corn silage, vetch hay, wet sugar beet pulp, and straw as roughages. Feeds were prepared as a total mixed ration. Cows in control, 1st and 2nd treatment groups received a diet containing 0, 250 and 500 g/d flaxseed, respectively. Adding flaxseed to the diet did not affect dry matter intake, milk yield, feed efficiency, or milk fat, lactose, and urea nitrogen (*P* > 0.05). However, it increased omega-3 fatty acids in milk and decreased the ratio of omega-6 to omega-3 fatty acids (*P* < 0.05). Results showed that adding different levels of flaxseed to the diets increased omega-3 fatty acids in milk without negatively affecting performance or fat percentages, making it suitable for producing functional milk.

## Introduction

With numerous studies in the field of human and animal nutrition, new developments have been achieved and as a result of these developments, people have become more conscious in the field of health. The high level of saturated fatty acids (SFA) in human diets increases the risk of cardiovascular diseases. Milk and dairy products generally contribute to 60% of SFA in human diets (Chilliard et al. 2007). By manipulating the diet, it is possible to decrease cholesterol levels and increase omega- 3 fatty acids (n3 FA) in milk, meat, and eggs. Omega- 3 fatty acids play an important role in brain development and the prevention of various disorders such as cardiovascular diseases and dementia. Since milk is an important food for human health, it is important to enrich milk in terms of omega- 3 fatty acids. Oilseeds are rich in polyunsaturated fatty acids and can be used to modify the milk fatty acid profile in dairy animals and further improve its nutritional value for humans (Kennelly 1996). The use of flaxseeds in ruminant diets can increase the level of fatty acids useful for human health in meat and dairy products. Milk fat contains more than 400 fatty acids and their isomers. Cow milk is rich in SFA, particularly C14:0 and C16:0, which are associated with increased plasma cholesterol levels and cardiovascular problems. However, it also contains smaller amounts of mono unsaturated fatty acids (MUFA), poly unsaturated fatty acids (PUFA), and omega- 3 fatty acids, which benefit human health (Suksombat et al. 2014). Flaxseed is an important oil seed that can be used as both an energy and protein source in ruminant diets and can be used in diets as raw, crushed, pressed, or its oil. Flaxseed is a rich source of plant lignans which are polyphenolic compounds having many biological activities including antioxidant, antitumor, and antiestrogenic properties. More than 95% of the total lignan in flaxseed is secoizolariciresinol diglycoside (SDG). Lignans in flaxseed are found in the hull, and ruminal microbiota convert SDG to mammalian lignans, enterodiol (ED), and enterolactone (EL). Flaxseed also contains small amounts of the plant lignan matairesinol, which is converted to EL by the colonic. Thus, in dairy cows fed flaxseed it is observed that milk is enriched in EL. EL-enriched milk being a dietary EL source for humans will become an excellent strategy to improve the effects of lignans on human health (Gagnon et al. 2009). Conjugated linoleic acid, one of the linoleic acid isomers, is found in dairy products and has anticarcinogenic, antiatherogenic, antiobesity, and immunomodulatory effects (McGuire and McGuire 2000). Flaxseed is a vegetable oil source rich in alpha-linolenic acid and it has been reported that the inclusion of flaxseeds in dairy diets reduced the concentration of short and medium-chain fatty acids in milk fat and increased the concentration of long-chain fatty acids (Mustafa et al. 2003; Petit 2003). For this reason, this study was conducted to determine the effects of adding different levels of flaxseed to the diets on the performance and milk fatty acid profile of dairy cows.

## Materials and methods

### Ethical considerations

Trials were carried out at a private enterprise and for the trials, the permission of the Provincial Directorate of Food, Agriculture and Livestock of the Ankara Governorship and ethical permission from the Ankara University Animal Experiments Local Ethics Committee were obtained (2018–7–58).

### Animal material

In this study, nine Holstein cows from a private enterprise in Ankara province were used. The cows were selected from a pool of 90 animals on the farm, based on average milk yield, lactation stage, parity, live weight, and age. Lactating cows in 1.5–3.0 months of lactation, which were in the 2nd or 3rd parity were used. Cows were housed individually.

### Feed material

The flaxseed used in the trial was obtained from the market. To crush the flaxseed, it was first crushed by mixing with concentrate feed (flaxseed: concentrate feed) in a 1:2 ratio. As feed material, two types of concentrate feeds and as roughage source corn silage, vetch hay, wet sugar beet pulp, and barley straw in the farm were used. The diet was prepared as a total mixed ration (TMR). TMR was fed 4 times a day. The TMR prepared in the experiment was formulated to meet the minimum requirement of the cows (NRC 2001). The TMR composition fed to the groups is given in Table 1. Flaxseed was included in the TMR for the groups at 0 (C), 0.25 (F), and 0.50 (2 F) kg/day, respectively.

**Table 1:** Composition of total mixed ration of groups (as fed), kg/d

Ingredients	C	F	2 F
Corn silage	9	9	9
Wet sugar beet pulp	14	14	14
Vetch hay	2.4	2.4	2.4
Barley straw	2.5	2.5	2.5
Flaxseed	0	0.25	0.50
Concentrate A (14.95% Crude Protein)	8.80	8.55	8.30
Concentrate B (20.32% Crude Protein)	4.30	4.30	4.30

C: Control group, F: group receiving 250 g/d flaxseed, 2 F: group receiving 500 g/d flaxseed

### Feeding and duration of experiment

The experiment was conducted between December 2017—March 2018. After placing cows in individual paddocks, cows were fed with control group TMR for 10 days. The experiment was designed as 3 periods, 3 groups, and 3 animals in each group according to the crossover trial order (Table 2). Each period lasted 21 days, of which 14 days was for adaptation and 7 days was for data collection. Between the periods, cows were fed with the control group diet for 10 days. Water was provided as ad libitum.

**Table 2:** Experimental design for groups according to period and animal number

	Animal number								
	1	2	3	4	5	6	7	8	9
Period 1	C	C	C	F	F	F	2 F	2 F	2 F
Period 2	2 F	2 F	2 F	C	C	C	F	F	F
Period 3	F	F	F	2 F	2 F	2 F	C	C	C

Groups: C: Control group, F: group receiving 250 g/d flaxseed, 2 F: group receiving 500 g/d flaxseed

### Data collection

The flaxseed-feed mixture was spread on the TMR which was given in the 4 th feeding of the day and thus, their consumption was ensured. The orts in the last 7 days of each period were collected every day in the morning and their amount was recorded. The ort was passed through a screen, and concentrate and roughage amounts were determined. Dry matter analyses of the ort were done and daily dry matter intake was calculated. In addition, the daily milk yield of the cows was recorded individually, at 7:00 and 19:00 in the last 7 days of each period. In the last three days of each period, morning and evening milk samples were taken to determine the dry matter, fat, protein and mineral contents of the milk with the help of a milk screening device (MilkoScan FT +, FOSS, Foss Alle 1, DK- 3400 Hilleroed-Denmark). The fatty acids composition of milk fat as fatty acid methyl esters (FAME) was analyzed with gas chromatography (Shimadzu GC, Shimadzu Co., Kyoto, Japan). The nutrient content of flaxseed, concentrate, and roughage used in the experiment was determined according to the methods reported in AOAC (2000). NDF and ADF analyses were done according to Georing and Van Soest’s (1970) method. In addition, the fatty acid composition of flaxseed was determined with gas chromatography (Shimadzu GC, Shimadzu Co., Kyoto, Japan).

### Statistical analysis

For Latin square design, a general linear model (GLM) with repeated measures (day 1–7 or day 1–7) was used to evaluate the main effects of group (G1, G2, G3) and period (P1, P2, P3), as well as their interaction, with the cow included as a covariance to account for individual variability. Milk yield was monitored during the seven days of the period, while milk composition and fatty acid profile were assessed daily during the last three days of each period. When a significant group, period, or interaction effect was detected, post hoc comparisons were conducted using the least significant difference (LSD) test. Data are presented as marginal means with their corresponding standard errors (SEM). Statistical analysis was done with SPSS (Inc., Chicago, IL, USA) program. As for the significance level, *P* < 0.05 level was chosen. (Dawson and Trapp 2001).

## Results and discussion

The chemical composition of flaxseed, the fatty acid profile of flaxseed, and the composition of feed ingredients used in the experiment are provided in Tables 3, 4, and 5, respectively. The flaxseed used in the study contained 25.60% crude protein and 38.10% ether extract (Table 3). Chemical composition and methylated fatty acids showed similarities to those reported in some studies (Suksombat et al. 2014; Oeffner et al. 2013).

**Table 3:** Chemical composition of flaxseed

Ingredients	%
Dry matter	91.75
Crude protein	25.60
Ether exctract	38.10
Crude fiber	7.70
Crude ash	2.90
Starch	4.57
ADF	14.70
NDF	23.90
Calcium	0.42
Phosphorus	0.44

*ADF* Acid Detergent Fiber, *NDF* Neutral Detergent Fiber

**Table 4:** Fatty acid profile of flaxseed (percent of total methylated fatty acid esters)

	Fatty acid	%
C12:0	Lauric acid	0.07
C14:0	Myristic acid	0.19
C16:0	Palmitic acid	5.84
C16:1 (n7)	Palmitoleic acid	0.07
C18:0	Stearic acid	3.13
C18:1 cis (n9)	Cis- 9 oleic acid	20.47
C18:2 cis (n6)	Linoleic acid	12.55
C20:0	Arachidonic acid	0.23
C18:3 (n6)	Gamma-linolenic acid	0.30
C20:1 (n9)	Cis- 11-eicosanoic acid (gondoic acid)	0.14
C18:3 (n3)	Alfa linolenic acid (ALA)	56.62
C22:0	Behenic acid	0.20
C24:0	Lignoseric acid	0.19
SFA	Saturated fatty acids	9.85
MUFA	Mono unsaturated fatty acids	20.68
PUFA	Poly unsaturated fatty acids	69.47
UFA	Unstaturated fatty acids	90.15
SFA/UFA	Ratio of saturated fatty acids/Unstaturated fatty acids	0.11
PUFA/SFA	Ratio of poly unsaturated fatty acids/Saturated fatty acids	7.05
n6	Omega- 6 fatty acids	12.85
n3	Omega- 3 fatty acids	56.62
n6/n3	Ratio of omega- 6/omega- 3 fatty acids	0.23

**Table 5:** Chemical composition of feed ingredients and concentrates, % (as fed)

Feed	Dry matter	Crude ash	Crude protein	Ether extract	Crude fiber	NDF	ADF
Corn silage	25.04	3.28	1.83	0.63	7.80	14.70	9.42
Wet sugar beet pulp	13.10	0.61	1.60	0.09	2.80	8.22	3.67
Vetch hay	90.85	7.88	14.30	1.65	20.92	44.70	31.05
Barley straw	87.98	7.10	2.33	0,42	45.02	70.97	50.20
Concentrate A	88.37	6.20	14.95	2.76	8.80	28.22	15.10
Concentrate B	89.94	9.40	20.32	3.06	12.90	35.20	15.05

*ADF* Acid Detergent Fiber, *NDF* Neutral Detergent Fiber

The effect of using different levels of flaxseeds in the diets of dairy cows on dry matter intake, milk yield, and feed conversion ratio (kg milk yield/kg dry matter intake) is given in Table 6. Adding flaxseeds at 0, 250 g, and 500 g per day did not affect dry matter intake, milk yield, and feed conversion ratio. In similar studies, it was shown that adding flaxseed or flaxseed oil to dairy cows’ and goats’ diets did not affect dry matter intake (Petit et al. 2004; Suksombat et al. 2014; Kholif et al. 2015; Huang et al. 2022). Benchaar et al. (2012) reported that the supplemental flaxseed oil at the levels of 2%, 3%, and 4% in dairy cow diets did not affect dry matter intake and nutrient digestibility. In a study conducted on beef cattle, Maddock et al. (2006) reported that adding flaxseed, crushed flaxseed, or grounded flaxseed at 8% of diet dry matter did not affect daily dry matter intake. Contrary to these findings, Martin et al. (2008) reported that in the lactation period supplementing dairy cows with 5.7% linseed oil decreased dry matter intake compared to the control group, but the inclusion of unprocessed or extruded flaxseed in the diet did not have any negative effect on dry matter intake. Chilliard (1993) concluded that fatty acid consumption has a direct inhibitory effect on dry matter intake through inhibition of rumino-reticular motility. The effect of unsaturated fat in flaxseed on dry matter intake generally depends on the amount of supplemental oil, the type of oil, and the roughage-concentrate ratio (Martin et al. 2008). Similar studies also reported that the inclusion of extruded flaxseed (Lerch et al. 2012), flaxseed (Cortes et al. 2010; Huang et al. 2022), and flaxseed oil (Caroprese et al. 2010) did not affect milk yield. Suksombat et al. (2014) observed that although in dairy cattle whole linseed supplementation increased the crude protein intake, no change was observed in milk yield. Contrary to these results, some researchers found that milk yield increased with the inclusion of flaxseed or flaxseed oil (Kholif et al. 2015), extruded linseed (Gomez-Cortes et al. 2009; Kholif et al. 2011), linseed oil (Benchaar et al. 2012) and whole grain linseed to the diet (Petit et al. 2004). Mach et al. (2013) found that the inclusion of 13% extruded flaxseeds in dairy diets increased milk yield. Despite these studies, some researchers found that the inclusion of flaxseed (Petit et al. 2005) and flaxseed oil (Brown et al. 2008; Mach et al. 2013) into the diet decreased milk yield. High levels of flaxseed oil consumption (greater than 5% of dry matter), decrease dry matter intake and digestibility due to impaired rumen functions and this may lead to a decrease in milk yield (Suksombat et al. 2014). In this study, since the oil consumption from flaxseed was below 5% of dry matter, no negative effect on dry matter intake and milk yield was observed.

**Table 6:** Effect of using different levels of flaxseed on milk yield, dry matter intake and feed conversion ratio*

	Milk yield, kg/d	Total dry matter intake, kg/d	FCR, kg milk/kg dry matter
Group			
1 (C)	26.81	20.03	1.34
2 (F)	26.92	20.01	1.35
3 (2 F)	27.03	20.01	1.35
Period			
1	26.64	20.01	1.33
2	27.14	20.00	1.36
3	26.98	20.03	1.35
Time, day			
1	26.22	20.04	1.31
2	27.33	20.03	1.37
3	27.44	20.01	1.37
4	26.44	19.98	1.32
5	26.82	19.99	1.34
6	26.89	20.01	1.34
7	27.30	20.04	1.36
Grand mean	26.92	20.01	1.35
SEM	0.406	0.019	0.020
Comparisons	*P*	*P*	*P*
Time	0.898	0.153	0.927
Group	0.975	0.898	0.968
Period	0.873	0.888	0.872
Group X Period interaction	0.301	0.838	0.312

^*^General linear model (GLM) with repeated measures (day 1–7) for the main effects of group and period, with the cow as covariance

*FCR* Feed conversion ratio, *SEM* standard error of mean

The effect of flaxseed on milk composition is shown in Table 7. There was no difference among the groups in terms of milk dry matter, fat, non-fat dry matter, lactose, and urea levels. The levels of milk fat, protein, and lactose were monitored through repeated measurements, and no significant differences were observed between periods or groups. While the mean level of total dry matter content of milk was similar across groups, a statistically significant decrease was observed from the first to the third period of the experiment. The mean non-fat DM content was lowest in the third period (*P* = 0.049). Additionally, the mean milk urea levels were highest in the second period of the study. These changes were not taken into account since every cow was included in each group, and every group was used in each period. Similar findings were reported by other researchers (Cortes et al. 2010; Petit and Cortes 2010; Lerch et al. 2012; Suksombat et al. (2014) who reported that supplementing diets with flaxseed or flaxseed oil did not affect milk composition, milk fat, and lactose yield. Weisbjerg et al. (2013) also found that supplementing lactating Holstein cows with 56 g ground rapeseed per kg of dry matter intake or 19 g ground flaxseed per kg of dry matter intake did not affect milk fat percentage and yield. Petit et al. (2004) reported that TMR having 9.7% whole flaxseed did not affect milk fat and milk protein percentage, but increased milk fat yield and milk protein yield. However, Huang et al. (2022) noted that whole flaxseed supplementation increased milk protein content, while the other components of milk showed no differences between groups. In contrast, supplemental DHA Gold alga with 3.1% flaxseed oil decreased the milk fat yield of dairy cows compared to the control group consuming a 3.1% protected saturated fat-containing control diet (Angulo et al. 2012). Dirandeh et al. (2013) indicated that supplementing dairy cows with 4.03% extruded flaxseed decreased milk fat percentage and yield did not affect the percentages of milk protein and lactose. Due to the high level of fat ingestion, the decrease in dry matter consumption and nutrient digestibility, especially fiber digestibility, may lead to a decrease in milk fat concentration (Martin et al. 2008). The addition of PUFA to starch-rich rations also leads to lower lipogenesis in the mammary tissue (Chilliard et al. 2007).

**Table 7:** Effect of using different levels of flaxseed on milk composition*

	Total DM, %	Fat %	Non-fat DM, %	Protein %	Lactose, %	Urea Nitrogen, mg/100 ml
Group						
1 (C)	12.75	3.54	9.26	3.47	5.64	13.42
2 (F)	12.78	3.54	9.26	3.57	5.56	13.15
3 (2 F)	12.72	3.58	9.19	3.44	5.58	13.08
Period						
1	12.95^a^	3.59	9.40^a^	3.59	5.67	12.75^b^
2	12.76^b^	3.53	9.26^a^	3.45	5.67	13.80^a^
3	12.54^c^	3.54	9.05^b^	3.44	5.45	13.11^b^
Time, day						
1	12.67^a^	3.52	9.19	3.49	5.52	13.10
2	12.76^ab^	3.55	9.24	3.48	5.61	13.20
3	12.82^b^	3.58	9.28	3.51	5.65	13.36
Grand mean	12.75	3.55	9.24	3.49	5.60	13.22
SEM	0.050	0.012	0.054	0.035	0.043	0.096
Comparisons	*P*	*P*	*P*	*P*	*P*	*P*
Time	0.064	0.083	0.247	0.634	0.225	0.146
Group	0.890	0.335	0.811	0.346	0.709	0.330
Period	0.014	0.078	0.049	0.186	0.087	0.001
Group X Period interaction	0.400	0.010	0.207	0.684	0.109	0.122

*DM* dry matter, *SEM* standard error of mean

^*^General linear model (GLM) with repeated measures (day 1–3) for the main effects of group and period, with the cow as covariance

Different superscript letters (a, b,c) within the same column indicate statistically significant differences among groups for the corresponding variable (*P* < 0.05)

The effect of flaxseed on milk fat fatty acid composition is given in Table 8. Among groups there was a difference in the following fatty acids C18:2 trans (n6) (*P* = 0.039), C18:3 (n3) (*P* < 0.001, C21:0 (*P* = 0.044), and C20:5 (n3) (*P* = 0.042). In addition, there was a significant difference among groups in terms of n3 (*P* < 0.001) fatty acids and n6/n3 (*P* < 0.001) fatty acids ratio. Omega- 3 fatty acids increased with the addition of flaxseed to the diet. With the addition of flaxseed, the reduction in the n6/n3 ratio indicates a decrease in the level of cis- 8,11,14-eicosationic acid (n6) and an increase in the level of stearic acid and alpha-linolenic acid (ALA, n3). There were significant differences among periods and notable interactions between group and period for some fatty acids (*P* > 0.05). However, these changes were not considered, as each cow was assigned to every group, and each group was utilized in every period.

**Table 8:** Effect of using different levels of flaxseed on fatty acid profile of milk as FAME, g/100 g fatty acid*

	C4:0	C6:0	C8:0	C10:0	C11:0	C12:0	C13:0	C14:0	C14:1 (n5)	C15:0	C16:0	C16:1 (n7)	C17:0	C17:1 (n7)	C18:0	C18:1-trans (n9)
Group																
1	1.26	1.52	1.33	3.89	0.064	4.67	0.130	14.29	1.03	1.45	30.73	1.70	1.02	0.179	9.99	0.404
2	1.26	1.50	1.32	3.86	0.069	4.62	0.136	13.87	1.08	1.48	30.16	1.69	1.04	0.175	10.57	0.426
3	1.38	1.59	1.38	4.03	0.066	4.78	0.126	14.30	1.03	1.35	30.36	1.70	1.01	0.178	10.50	0.401
Period																
1	1.40	1.66	1.43	4.20	0.082^a^	5.12^a^	0.142	14.71	1.13^a^	1.46	30.85	1.73	1.01^ab^	0.179	9.78^a^	0.359^a^
2	1.24	1.50	1.34	3.86	0.063^b^	4.53^b^	0.123	13.68	0.97^b^	1.34	29.50	1.64	0.98^a^	0.174	10.79^b^	0.353^a^
3	1.25	1.45	1.27	3.71	0.055^b^	4.43^b^	0.127	14.07	1.03^ab^	1.48	30.90	1.72	1.07^b^	0.178	10.50^ab^	0.519^b^
Time, day																
1	1.31	1.52	1.32	3.85	0.067	4.66	0.132	13.85	1.03	1.46	30.51	1.69	1.03	0.176	10.42	0.398
2	1.27	1.52	1.34	3.90	0.067	4.65	0.130	13.82	1.05	1.38	29.51	1.70	1.04	0.176	10.24	0.433
3	1.31	1.57	1.38	4.03	0.066	4.77	0.130	14.79	1.05	1.44	30.22	1.70	0.99	0.180	10.40	0.400
Grand mean	1.30	1.54	1.35	3.92	0.067	4.69	0.131	14.15	1.04	1.43	30.42	1.70	1.02	0.177	10.35	0.410
SEM	0.034	0.035	0.031	0.099	0.003	0.103	0.005	0.182	0.025	0.032	0.294	0.037	0.011	0.006	0.149	0.016
Comparisons	*P*	*P*	*P*	*P*	*P*	*P*	*P*	*P*	*P*	*P*	*P*	*P*	*P*	*P*	*P*	*P*
Time	0.434	0.270	0.217	0.356	0.352	0.500	0.163	0.497	0.722	0.491	0.049	0.939	0.691	0.966	0.455	0.689
Group	0.272	0.530	0.759	0.770	0.854	0.811	0.681	0.567	0.653	0.244	0.726	0.980	0.520	0.969	0.242	0.789
Period	0.144	0.062	0.120	0.146	0.016	0.030	0.229	0.093	0.049	0.184	0.118	0.568	0.023	0.947	0.037	0.001
Group X Period Interaction	0.553	0.598	0.607	0.554	0.232	0.454	0.380	0.371	0.339	0.091	0.183	0.086	0.033	0.151	0.523	0.274

	C18:1 cis (n9)	C18:2 trans (n6)	C18:2-cis (n6)	C20:0	C18:3 (n6)	C20:1 (n9)	C18:3 (n3)	C21:0	C20:2 (n6)	C22:0	C20:3 (n6)	C23:0	C20:4 (n6)	C22:2	C24:0	C20:5 (n3)
Group																
1	22.34	0.178^x^	2.76	0.184	0.025	0.059	0.198^x^	0.010^x^	0.012	0.049	0.189	0.277	0.025	0.011	0.026	0.017^x^
2	22.60	0.221^y^	2.69	0.186	0.026	0.069	0.323^y^	0.016^y^	0.013	0.054	0.184	0.271	0.029	0.013	0.029	0.027^y^
3	21.98	0.199^xy^	2.55	0.178	0.018	0.054	0.296^y^	0.010^x^	0.011	0.043	0.168	0.251	0.021	0.011	0.019	0.019^xy^
Period																
1	20.92	0.176^a^	2.70^a^	0.154^a^	0.019	0.063	0.217^a^	0.011	0.010	0.030^a^	0.160	0.239	0.019	0.012	0.017	0.017^a^
2	23.40	0.231^b^	3.00^a^	0.190^b^	0.024	0.059	0.360^b^	0.012	0.014	0.058^b^	0.189	0.291	0.024	0.011	0.027	0.028^b^
3	22.60	0.191^a^	2.30^b^	0.204^b^	0.026	0.061	0.240^a^	0.013	0.011	0.058^b^	0.192	0.270	0.032	0.012	0.029	0.018^a^
Time, day																
1	23.31	0.199	2.67	0.186	0.024	0.058	0.269	0.009	0.012	0.044	0.183	0.269	0.023	0.011	0.022	0.020
2	22.34	0.208	2.70	0.176	0.021	0.061	0.284	0.013	0.012	0.050	0.178	0.256	0.026	0.012	0.024	0.020
3	22.26	0.191	2.64	0.183	0.024	0.064	0.264	0.014	0.011	0.053	0.180	0.274	0.026	0.012	0.027	0.023
Grand mean	22.30	0.199	2.66	0.183	0.023	0.061	0.272	0.012	0.012	0.049	0.180	0.266	0.025	0.012	0.024	0.021
SEM	0.395	0.006	0.065	0.004	0.002	0.003	0.009	0.001	0.001	0.003	0.007	0.008	0.001	0.001	0.002	0.002
Comparisons	*P*	*P*	*P*	*P*	*P*	*P*	*P*	*P*	*P*	*P*	*P*	*P*	*P*	*P*	*P*	*P*
Time	0.875	0.975	0.328	0.700	0.319	0.474	0.615	0.154	0.562	0.819	0.575	0.302	0.418	0136	0.666	0.556
Group	0.814	0.039	0.427	0.756	0.148	0.158	< 0.001	0.044	0.583	0.299	0.524	0.435	0.076	0.199	0.196	0.042
Period	0.056	0.006	0.001	< 0.001	0.299	0.815	< 0.001	0.783	0.191	0.001	0.177	0.063	0.003	0.936	0.104	0.020
Group X Period Interaction	0.192	0.066	0.988	0.337	0.438	0.162	0.012	0.440	0.726	0.245	0.040	0.488	0.241	0.459	0.732	0.646

	SFA	MUFA	PUFA	UFA	SFA/ UFA	PUFA/ SFA	n6	n3	n6/n3
Group									
1	70.88	25.71	3.41	29.12	2.49	0.048	3.20	0.214^x^	15.53^x^
2	70.45	26.03	3.52	29.55	2.42	0.050	3.17	0.350^y^	9.82^y^
3	71.37	25.34	3.30	28.63	2.54	0.046	2.98	0.315^y^	10.36^y^
Period									
1	72.29	24.38	3.33	27.71	2.67	0.046^a^	3.10^a^	0.233^a^	14.23^a^
2	69.52	26.59	3.89	30.48	2.32	0.056^b^	3.50^b^	0.387^b^	10.47^b^
3	70.88	26.10	3.02	29.12	2.49	0.043^a^	2.76^a^	0.258^a^	11.01^b^
Time,day									
1	70.93	25.66	3.41	29.08	2.46	0.048	3.13	0.287	11.82
2	70.78	25.76	3.46	29.22	2.50	0.049	3.15	0.304	11.55
3	72.98	25.65	3.37	29.02	2.70	0.048	3.08	0.287	12.33
Grand mean	70.90	25.69	3.41	29.10	2.48	0.048	3.12	0.293	11.90
SEM	0.454	0.423	0.076	0.453	0.058	0.001	0.070	0.009	0.316
Comparisons	*P*	*P*	*P*	*P*	*P*	*P*	*P*	*P*	*P*
Time	0.834	0.908	0.405	0.833	0.588	0.579	0.324	0.658	0.186
Group	0.715	0.802	0.489	0.714	0.676	0.461	0.398	< 0.001	< 0.001
Period	0.071	0.111	0.001	0.071	0.075	0.001	0.002	< 0.001	< 0.001
Group X Period Interaction	0.186	0.182	0.714	0.186	0.185	0.544	0.857	0.014	0.030

C4:0 = butyric acid; C6:0 = caproic acid; C8:0 = caprylic acid; C10:0 = capric acid; C11:0 = undecanoic acid; C12:0 = lauric acid; C13:0 = tridecanoic acid; C14:0 = myristic acid; C14:1(n5) = myristoleic acid; C15:0 = pentadecanoic acid; C16:0 = palmitic acid; C16:1(n7) = palmitoleic acid; C17:0 = heptadecanoic acid (margaric acid); C17:1(n7) = cis- 10-heptadecanoic acid; C18:0 = stearic acid; C18:1-trans (n9) = elaidic acid; *FAME* Fatty acid methyl esters, *SEM* standard error of mean

Different superscript letters (a, b) within the same column indicate statistically significant differences among groups for the corresponding variable (*P* < 0.05)

C18:1-cis(n9) = cis- 9-oleic acid, C18:2-trans (n6) = linoelaidic acid, C18:2-cis (n6) = linoleic acid, *C20:0* = *arachidic acid, C18:3 (n6)* = *gamma-linolenic acid, C20:1(n9)* = *cis- 11-eicosenoic acid (gondoic acid),* C18:3(n3) = alfa linolenic acid (ALA), C21:0 = heneicosanoic acid; C20:2(n6) = eicosadieonic acid; C22:0 = behenic acid; C20:3(n6) = cis- 8,11,14-eicosatrieonic acid; C23:0 = tricosanoic acid; C20:4(n6) = arachidonic acid; C22:2(n6) = cis- 13,16-docosadieonic acid; C24:0 = lignoceric acid; C20:5(n3) = Eicosapentaenoic acid (EPA)

*SFA* saturated fatty acids, *MUFA* mono unsaturated fatty acids, *PUFA* poly unsaturated fatty acids, *UFA* unsaturated fatty acids, *FAME* Fatty acid methyl esters, *SEM* standard error of mean

*General linear model (GLM) with repeated measures (day 1–3) for the main effects of group and period, with the cow as covariance

Different superscript letters (a, b, and x, y) within the same column indicate statistically significant differences among groups for the corresponding variable (*P* < 0.05)

**S**imilar to the results from this study, Kholif et al. (2015) reported that a goat diet supplemented with flaxseed or flaxseed oil increased ALA level in milk, which led to a decrease in the ratio of n6/n3 fatty acids. Low n6/n3 ratio in foods is known to prevent some diseases in humans (Connor 2000). Suksombat et al. (2014) reported that adding flaxseed or flaxseed oil to the diets increased EPA, DHA, n3 fatty acids and UFA levels in milk (*P* < 0.01), and decreased SFA level and n6/n3 ratio. Some other researchers also found similar findings (Lerch et al. 2012; Mach et al. 2013; Weisbjerg et al. 2013). In some other studies, it was found that supplemental flaxseed or flaxseed oil increased milk percentage, MUFA, PUFA, and total UFA percentage, and decreased SFA percentage (Caroprese et al. 2010; Petit and Cortes 2010; Lerch et al. 2012; Suksombat et al. 2014). Petit et al. (2004) reported that adding 9.7% whole flaxseed in TMR, significantly decreased SFA, n6 fatty acids, and n6/n3 ratio, and significantly increased UFA and n3 fatty acid concentration. Similar to the present study, Huang et al. (2022) also observed that flaxseed supplementation increased milk ALA content and reduced the ratio of n6 to n3. In a study conducted on beef cattle, Maddock et al. (2006) found that whole flaxseeds, crushed flaxseeds, and ground flaxseeds constituting 8% of dry matter in the ration increased ALA and n3 fatty acids levels and decreased n6 fatty acid levels in meat.

## Conclusions

Results showed that adding flaxseed to dairy diets did not cause a significant difference in dry matter intake, milk yield, and feed efficiency. There were no differences among the groups in terms of milk dry matter, fat, non-fat dry matter, lactose, and urea levels. Adding flaxseed to the ration increased omega 3 fatty acids and decreased n6/n3 ratio in milk fat. Therefore, the results suggest that incorporating flaxseed into the diets can contribute to the production of functional milk.

## Data Availability

The data that support the findings of this study are available from the corresponding author, upon reasonable request.
